# Motor and Non-motor Symptoms Associated With Exercise Behavior in Parkinson's Disease Patients: Factors Differ Between Patients With and Without Postural Instability

**DOI:** 10.3389/fneur.2021.772391

**Published:** 2021-11-30

**Authors:** Joomee Song, Jinyoung Youn, Young Eun Huh, Jun Kyu Mun, Jong Hyeon Ahn, Dongyeong Lee, Woo Young Shin, Jin Whan Cho

**Affiliations:** ^1^Department of Neurology, Samsung Medical Center, Sungkyunkwan University School of Medicine, Seoul, South Korea; ^2^Neuroscience Center, Samsung Medical Center, Seoul, South Korea; ^3^Department of Neurology, CHA Bundang Medical Center, CHA University, Seongnam, South Korea

**Keywords:** Parkinson's disease, exercise, postural instability, PASE, physical activity

## Abstract

**Background:** Exercise is an important treatment for Parkinson's disease (PD). Therefore, recognizing determinants of exercise behavior for PD based on disease stage is essential. We sought to find whether the determinants differ based on presence of postural instability (PI), which is indicative of disease stage in PD.

**Methods:** We enrolled patients at Samsung Medical Center from September 2019 to November 2020, who had the ability to perform exercise [modified Hoehn and Yahr (HY) stage ≤ 3]. All the motor and non-motor symptoms were investigated. The exercise of the PD patients was evaluated using the Physical Activity Scale of the Elderly (PASE)-leisure score. We classified patients into PD without PI (HY stage 1 – 2) and PD with PI (HY stage 2.5 – 3) groups. Multivariate linear regression was performed using backward elimination in each group to determine factors associated with PASE-leisure score.

**Results:** A total of 233 patients were enrolled. In the PD without PI group (*n* = 177), the positive determinant of exercise was Activities-Specific Balance Confidence (ABC) score (β = 0.142, *p* = 0.032), and the negative determinants were fatigue score (β = −0.228, *p* = 0.018), female (β = −6.900, *p* = 0.016) and currently employed status (β = −6.072, *p* = 0.046). In the PD with PI group (*n* = 56), the positive determinant was non-motor symptom scale (NMSS) score (β = 0.221, *p* = 0.017) and disease duration (β = 1.001, *p* = 0.036), while the negative determinants were UPDRS part 3 score (β = −0.974, *p* < 0.001), UPDRS part 4 score (β = −2.192, *p* = 0.002), and age (β = −1.052, *p* < 0.001).

**Conclusion:** Different motor and non-motor symptoms were associated with the exercise in PD patients with and without PI. When encouraging PD patients to exercise, personalized and different strategies should be applied based on the presence of PI.

## Introduction

Exercise as a treatment for Parkinson's disease (PD) is becoming primary, rather than supplementary, in the management of PD patients. In many studies, higher level of physical activity and regular exercise were associated with less progression of motor and cognitive dysfunction in PD patients (1, 2). Furthermore, physical inactivity is associated with greater complications such as aspiration pneumonia or bedsores in advanced PD (3). Therefore, inspiring and motivating PD patients to engage in exercise could play an important role in improving their prognosis. First, clarifying determinants of exercise behavior in PD patients is necessary. In normal elderly population studies, self-efficacy, educational level, and other psychiatric perspectives were reported as determinant factors; however, consensus is lacking (4).

PD patients are a distinctive group with motor and non-motor symptoms and can have different determinants of exercise behavior compared with normal elderly people. Reportedly, motor and non-motor symptoms significantly limit activities of daily living (ADL) and physical activity of PD patients (5–7); thus, those symptoms likely play a significant role in determining exercise behavior of PD patients. However, a few determinants reportedly associated with exercise amount in PD patients were self-efficacy or other psychiatric perspectives or demographics (8, 9), which did not show any difference from results in previous normal elderly population studies. Motor or non-motor symptoms of PD were not investigated properly in former studies, and the parkinsonian symptoms as determinants of exercise behavior are not well-known.

In particular, postural instability (PI), which develops as the disease progresses, significantly contributes to both physical inactivity and decreased ADL compared with other motor symptoms (5, 10). Consequently, advanced PD patients who have PI have different exercise behaviors and determinants from those without PI. Therefore, using a different strategy to encourage exercise based on the presence of PI in PD patients would result in better exercise behavior and prognosis. In the present study, the specific motor or non-motor symptoms of PD that could determine exercise behavior and whether those determinants would differ between PD without PI and PD with PI groups were investigated.

## Materials and Methods

### Subjects

The present study included PD patients diagnosed at Samsung Medical Center from September 2019 to November 2020, who could exercise [modified Hoehn and Yahr (HY) stage ≤ 3] (11). PD was diagnosed according to the U.K. Parkinson's Disease Society Brain Bank criteria (12).

We excluded patients with Parkinson-plus syndrome (multiple systemic atrophy, progressive supranuclear palsy, and corticobasal syndrome), secondary parkinsonism (vascular parkinsonism, drug-induced parkinsonism, or normal pressure hydrocephalus), or structural brain lesions, including stroke or tumor, cardiopulmonary, musculoskeletal problems, or other neurological conditions (e.g., myelopathy, known neuropathy, chronic vestibular dysfunction) that preclude exercise. Patients with dementia [Korean-Mini Mental Status Exam (K-MMSE) score ≤ 20] (13, 14) were excluded to maintain reliability of exercise reports and major psychiatric diseases requiring medical treatment, including major depressive disorder, bipolar and related disorders, and schizoaffective disorders diagnosed according to DSM-V criteria (15).

Finally, patients were classified into the PD without PI (modified HY stage 1–2) and PD with PI (modified HY stage 2.5–3) groups.

### Predictors of Exercise Behavior

Demographic information of age, sex, educational level, current employment status, and presence of comorbidities was collected, which reportedly affected exercise behavior in PD patients or healthy elderly subjects (4).

Regarding motor symptoms, Unified Parkinson's Disease Rating Scale (UPDRS) part 3 during “on” state (11), UPDRS part 4, and Freezing of Gait Questionnaire (FOGQ) (16) were assessed in each patient.

Overall, non-motor symptoms were evaluated using a non-motor symptom scale (NMSS) (17). Each of the non-motor symptoms was evaluated in detail. Neuropsychiatric features, including anxiety and depressive mood, were evaluated using the Beck Anxiety Inventory (BAI) (18) and Beck Depression Index (BDI) (19). Parkinson's Fatigue Scale (20) was used to assess fatigue. Excessive daytime sleepiness and insomnia were evaluated using Parkinson's Disease Sleep Scale (PDSS) (21), and autonomic dysfunctions were assessed using Scales for Outcomes in Parkinson's Disease–Autonomic Dysfunction (SCOPA-AUT) (22).

### Measurements of Exercise Behavior: Physical Activity Scale of the Elderly (PASE) Score

The exercise behavior of the PD patients was evaluated using the Korean version of the Physical Activity Scale of the Elderly (PASE) (23–25), a validated, self-reported questionnaire assessing the frequency, intensity, and duration of activity over the prior week. The PASE is composed of three sections (leisure exercise, work/volunteering, and household chores), and higher PASE scores indicate greater physical activity, with scores ranging from 0 to 365 in the validated sample. The leisure exercise (PASE-leisure) section includes all types of sports activity; only the PASE-leisure score was used in the present study for exercise analysis.

### Statistical Analysis

Data are presented as mean ± standard deviation (SD) or number (%). Demographics and clinical data were compared between PD with PI and PD without PI groups using Student's *t*-test or Mann-Whitney *U* test for continuous variables. To determine a model with factors that explains PASE-leisure score, multivariate linear regression analyses were performed in PD with PI and PD without PI groups. All variables were included in the multivariate linear analysis, and backward elimination was performed; the reference for elimination was when F probability was or/and above 0.1 and reference for entrance was when F probability or/below 0.05. The backward elimination was done until all the factors satisfied the reference value for the F probability and had significant p-value (< 0.05) in the model. All demographic parameters in this study were previously reported to be associated with exercise behavior. The variance inflation factor (VIF) among the variables was calculated to detect multi-co-linearity in multivariate analysis. All *p*-values and 95% confidence intervals (CIs) for the odds ratio (OR) were corrected using Bonferroni's method due to multiple testing after controlling for potential confounders. IBM SPSS Statistics for Windows (Version 27.0. IBM Co.; Armonk, NY, USA) and SAS version 9.4 (SAS Institute, Cary, NC, USA) were used for statistical analyses.

## Results

### Demographic, Clinical, and Exercise Characteristics of Recruited Subjects

A total of 233 PD patients was enrolled. The mean age was 67.7 ± 8.75 years and 114 (48.9 %) were males. Among subjects, 177 had PD without PI and 56 had PD with PI. The subject demographics, clinical characteristics, and exercise characteristics are summarized and compared in Table 1. All the motor symptom-related scores including UPDRS parts 3 and 4 and FOGQ were significantly higher in the PD with PI subjects. The Activities-Specific Balance Confidence (ABC) score was significantly higher in PD without PI subjects. Regarding non-motor symptoms, total NMSS, SCOPA-AUT, and PDSS scores were significantly different between the two groups. Demographics of disease duration, MMSE, comorbidities, and employment status were significantly different between groups. PD without PI subjects showed higher PASE-leisure score than PD with PI subjects but without statistical significance (*p* = 0.052).

**Table 1:** Demographics and clinical characteristics of patients.

	**Total (*n* = 233)**	**Without PI (*n* = 177)**	**With PI (*n* = 56)**	**p-value**
**Demographics**				
Age	67.27 ± 8.75	66.70 ± 8.38	69.05 ± 9.69	0.060
Sex, male	114 (48.9)	88 (49.7)	26 (46.4)	0.668
BMI	24.10 ± 3.12	24.19 ± 3.07	23.81 ± 3.27	0.600
MMSE	27.48 ± 2.60	27.73 ± 2.46	26.66 ± 2.87	0.009^*^
Years of education	12.20 ± 4.36	12.45 ± 4.02	11.41 ± 5.26	0.306
Currently employed	114 (48.9 %)	66 (37.3)	11 (19.6)	0.014^*^
Comorbidities	1 (0–2)	1 (0–1)	0 (0–1)	0.038^*^
Disease duration (years)	6.94 ± 5.39	6.02 ± 4.98	9.84 ± 5.66	<0.001^*^
**Parkinsonism, motor symptoms**				
HY stage (1/1.5/2/2.5/3)	65 (27.9)/11 (4.7)/101 (43. 3)/31 (13.3)/25 (10.7)			
UPDRS part 3	15.35 ± 8.52	13.39 ± 7.03	21.57 ± 9.81	<0.001^*^
UPDRS part 4	3.15 ± 4.25	2.30 ± 3.13	5.84 ± 5.94	<0.001^*^
FOGQ	4.19 ± 5.18	3.19 ± 4.13	7.34 ± 6.73	<0.001^*^
ABC score	78.00 ± 25.30	80.72 ± 24.00	69.39 ± 27.54	<0.001^*^
**Parkinsonism, non-motor symptoms**				
NMSS	36.52 ± 30.31	32.32 ± 28.83	49.82 ± 42.29	0.002^*^
BDI	11.77 ± 9.21	11.14 ± 8.55	13.79 ± 10.87	0.162
BAI	10.45 ±9.93	9.54 ± 8.29	13.36 ± 13.57	0.177
PDSS	110.91 ± 30.31	113.48 ± 28.71	102.79 ± 33.89	0.038^*^
SCOPA-AUT	17.63 ± 9.93	16.25 ± 9.16	21.98 ± 11.08	<0.001^*^
Fatigue scale	40.68 ± 16.36	39.87 ± 16.20	43.25 ± 16.75	0.190
**Exercise behavior**				
PASE-leisure	23.24 ± 19.24	24.29 ± 18.90	19.89 ± 20.10	0.052

*Data are presented as mean ± standard deviation (SD), median (interquartile range), or n (%); BMI, body mass index; MMSE, Mini Mental Status Examination; HY, Hoehn and Yahr stage; UPDRS, Unified Parkinson's Disease Sale; FOGQ, Freezing of Gait Questionnaire; ABC, Activities-Specific Balance Confidence; BDI, Beck Depression Inventory; BAI, Beck Anxiety Inventory; PDSS; Parkinson's Disease Sleep Scale; SCOPA-AUT, Scales for Outcomes in Parkinson's disease-Autonomic Dysfunction. PI, postural instability; NMSS, non-motor symptom scale for Parkinson's disease; PASE, Physical Activity Scale of the Elderly.*

* *Variables with p < 0.05 in the group comparison*.

### Factors Associated With PASE Score

The multivariate linear regression results for each group are summarized in Table 2. In the PD without PI group, the positive determinant of exercise was ABC score [β = 0.142, standard error of β (SE) = 0.066, *p* = 0.032], and the negative determinants were fatigue score (β = −0.228, *SE* = 0.096, *p* = 0.018), female (β = −6.900, *SE* = 2.826, *p* = 0.016), and currently employed status (β = −6.072, *SE* = 3.018, *p* = 0.046).

**Table 2:** Multivariate linear regression analyses of the associations of PASE score and clinical factors.

		**Multivariate analysis**			
	**Variables**	**β**	***SE***	***p-value***	**VIF**
**Without PI**
Demographics	Female sex	−6.900	2.826	0.016	1.122
	Currently employed	−6.072	3.018	0.046	1.198
Motor symptoms	ABC score	0.142	0.066	0.032	1.402
Non-motor symptoms	Fatigue scale	−0.228	0.096	0.018	1.349
**With PI**
Demographics	Age	−1.052	0.294	<0.001	1.499
	Disease duration	1.001	0.464	0.036	1.275
Motor symptoms	UPDRS part 3	−0.974	0.259	<0.001	1.194
	UPDRS part 4	−2.192	0.676	0.002	2.987
Non-motor symptoms	NMSS	0.221	0.090	0.017	2.657

*Data are presented as mean ± standard deviation (SD), median (interquartile range), or n (%); SE, standard error of β; VIF, variance inflation factor; ABC, Activities-Specific Balance Confidence; UPDRS, Unified Parkinson's Disease Sale; NMSS, non-motor symptom scale*.

In PD with PI subjects, the positive determinants were NMSS (β = 0.221, *SE* = 0.090, *p* = 0.017), and disease duration (β = 1.001, *SE* = 0.464, *p* = 0.036), and the negative determinants were UPDRS part 3 score (β = −0.974, *SE* = 0.259, *p* < 0.001), UPDRS part 4 (β = −2.192, *SE* = 0.676, *p* = 0.002), and age (β = −1.052, *SE* = 0.294, *p* < 0.001).

## Discussion

Exercise is a promising alternative treatment for PD and should be included in disease management for PD patients. To strategically encourage exercise in PD patients, knowing the determinants of exercise behavior based on disease stage is necessary. This is the first study which addresses the determinants of exercise behaviors based on the presence of PI in PD patients. PD patients with and without PI showed different determinants of exercise in the present study, which suggests different approaches are needed to increase exercise in PD patients.

In PD without PI patients, the positive predictor was self-confidence in postural stability (ABC score), which is modifiable. Therefore, early intervention to increase confidence of postural stability in early PD would result in better exercise behavior and improved prognosis in the entire PD population. PD patients who have discrepancy between objective PI and ABC score (26) would benefit most from exercise resulting in improved prognosis. The negative predictor in PD without PI patients was fatigue, which is considered one of the most disabling non-motor symptoms in PD, manifesting in the beginning of the disease and lowering the quality of life (27). Therefore, assessing and managing fatigue specifically is important for improving quality of life and modifying exercise behavior, which can lead to better prognosis.

In PD with PI patients, “on” state motor symptoms (UPDRS part 3, 4), which likely cannot be modified, were negative determinants of PASE-leisure score. Advanced PD patients with PI suffer severe motor symptoms or motor fluctuations, which are challenging to manage with pharmacological intervention. Therefore, motor symptoms are more likely to affect both the motivation to exercise and maintenance of exercise in advanced PD than in early PD. Notably, NMSS was a positive predictor of PASE-leisure score in PD with PI patients. A possible explanation is that non-motor symptoms usually do not respond to pharmacological treatment in PD patients, and exercise is an alternative solution. Many patients report relief of various NMSS after exercising, although the effect can be transient (28–30), which could motivate patients to exercise.

Regarding socioeconomic determinants, currently employed status was a negative determinant in PD without PI patients, which could be explained by lack of time to exercise and fatigue from working. This is concerning for PD patients under 65 years of age who are part of the productive population and mostly working. Female gender was another negative predictor of exercise in PD without PI patients, which is a similar result to a previous study with healthy elderly subjects; females showed significantly lower PASE-leisure score than males (23, 25, 31). Female PD patients, had less opportunity for the education in Korea of 1950s and 1960s compared to male patients. Considering educational years was reported to be a positive factor of exercise amount in a study in normal elderly (4), our study's result is comprehensible. Also, in Asian culture, female is the main gender who does the most of housework. Naturally, as a female, it is hard to have time to exercise like currently employed people are. Stronger encouragement for exercise in those patients is needed.

In PD with PI patients, there were no socioeconomic predictors other than age and disease duration, which are indicators of disease progression. Notably, although age was a negative predictor, disease duration was a positive predictor. This result indicates that subjects who develop PD earlier in life are likely to exercise more. Another possible explanation is that subjects who exercise more survived longer or had better prognosis resulting in longer disease duration, which appears to contradict the result showing UPDRS part 3 as a negative predictor of exercise. However, the lower correlation of MDS-UPDRS score with disease duration has been reported in a previous study (32). Considering that our study population of PD with PI included patients with modified HY stage 2.5–3, the explanation that patients with a long disease duration have a better prognosis correlates with the positive association between exercise and disease duration in the present study.

The result of the present study may show the importance of early intervention to change exercise behavior in early PD patients without PI. Exercise behavior of PD with PI patients is affected mainly by disease-related factors such as general motor and non-motor symptoms and disease duration, which is relatively unmodifiable. Conversely, in PD without PI patients, exercise behavior is affected by self-confidence in postural stability, fatigue, and employment status, which are modifiable. Therefore, early management of those factors to encourage exercise in early PD patients is needed for better prognosis.

As the COVID-19 pandemic was prevalent during this study period, the patients' exercise behavior has been influence by change of society during the pandemic such as social distancing. In a previous study, the exercise amount was reduced in PD during the COVID-19 pandemic (33). Therefore, the pandemic itself could be a factor of exercise behavior. However, even though the general exercise amount could have been reduced during the COVID-19 pandemic, the determinants for the exercise behavior would have not changed much. It would be an interesting study theme whether the determinants change significantly after the pandemic.

The present study had several limitations. First, due to the cross-sectional design, the causation between factors mentioned and exercise behavior is suspected but not proven. Second, this was a single-center, single-ethnicity study that could show biased results associated with social status or ethnicity. In addition, the number of PD patients with PI was relatively small compared with that of patients without PI, which could decrease the power of analysis. However, the power was 0.974 (*R*^2^ = 0.331) when the multivariate regression model of PD with PI was statistically analyzed. Moreover, the proportion of the patients with PI and without PI of our study correspond with reported epidemiology of PD, which shows PD with PI is almost one third of patients without PI (32). Third, patient reports were used to investigate exercise behavior, which have the risk of reporting and recall biases. However, the PASE was used, which is a validated measurement of physical activity that has been shown to correlate well with objective measures of physical activity (23). We did not include the socioeconomic determinants such as income status and condition of marriage which can be related factors for exercise behavior. To investigate the accurate financial status of patients, we have to gather all the information about real estate, movables, as well as their family's financial status. In a clinical setting, investigation of income can be significantly inaccurate because physician should depend entirely on interview with patients, not on written data. Regarding marriage status, many patients were reluctant to open about their marriage status. Still, we included major socioeconomic factors like age, sex, educational background and employment status which has been reported to be associated with exercise behavior in previous studies. Finally, patients who had major psychiatric diseases requiring medical treatment were excluded, which limits the generalizability of the results for PD patients with more severe psychiatric problems.

In conclusion, when encouraging PD patients to exercise, strategies can differ based on the presence of PI; strengthening confidence of postural stability and relieving fatigue are important factors in exercise behavior of PD without PI patients. However, parkinsonism significantly affects exercise behavior in PD with PI patients. Therefore, for patients without PI, ensuring their postural stability while encouraging exercise in clinic would be a strategy. Also, regular exercise and gradual increment in intensity of exercise should be recommended. On the other hand, for patients with PI, best medical treatment that can lower the UPDRS part 3 and 4 as much as possible is required. Considering that there are symptoms which do not respond to medication (i.e., PI), recommending exercises that has lower risk of falling can be another strategy. Early intervention to modify the relatively modifiable factors in early PD without PI patients would lead to better exercise behavior and prognosis.

## Data Availability Statement

The raw data supporting the conclusions of this article will be made available by the authors, without undue reservation.

## Ethics Statement

The studies involving human participants were reviewed and approved by Institutional Review Board (IRB) of Samsung Medical Center (IRB number: 2020-09-214). Written informed consent for participation was not required for this study in accordance with the national legislation and the institutional requirements.

## Author Contributions

JS conceived and designed the study and acquired, analyzed and interpreted data, and drafted and revised the manuscript. JC conceived and designed, and supervised the study, interpreted data, and drafted and revised the manuscript for intellectual content. YH and JY revised the manuscript for intellectual content. JM and JA acquired and interpreted data. DL and WS acquired data. All authors contributed to the article and approved the submitted version.

## Conflict of Interest

The authors declare that the research was conducted in the absence of any commercial or financial relationships that could be construed as a potential conflict of interest.

## Publisher's Note

All claims expressed in this article are solely those of the authors and do not necessarily represent those of their affiliated organizations, or those of the publisher, the editors and the reviewers. Any product that may be evaluated in this article, or claim that may be made by its manufacturer, is not guaranteed or endorsed by the publisher.
